# Inhibition of the key metabolic pathways, glycolysis and lipogenesis, of oral cancer by bitter melon extract

**DOI:** 10.1186/s12964-019-0447-y

**Published:** 2019-10-21

**Authors:** Subhayan Sur, Hiroshi Nakanishi, Colin Flaveny, Joseph E. Ippolito, Jane McHowat, David A. Ford, Ratna B. Ray

**Affiliations:** 1 Department of Pathology, Saint Louis University, 1100 South Grand Boulevard, St. Louis, MO 63104 USA; 2 Department of Pharmacology and Physiology, Saint Louis University School of Medicine, St. Louis, MO USA; 3 Mallinckrodt Institute of Radiology, Washington University in Saint Louis School of Medicine, Saint Louis, MO USA; 4 Biochemistry and Molecular Biology, Saint Louis University, Saint Louis, MO USA

**Keywords:** Bitter melon extract, Oral cancer, Glycolysis, Lipid metabolism, Phosphatidylcholine, Phosphatidylethanolamine, ROS

## Abstract

**Background:**

Metabolic reprogramming is one of the hallmarks of cancer which favours rapid energy production, biosynthetic capabilities and therapy resistance. In our previous study, we showed bitter melon extract (BME) prevents carcinogen induced mouse oral cancer. RNA sequence analysis from mouse tongue revealed a significant modulation in “Metabolic Process” by altering glycolysis and lipid metabolic pathways in BME fed group as compared to cancer group. In present study, we evaluated the effect of BME on glycolysis and lipid metabolism pathways in human oral cancer cells.

**Methods:**

Cal27 and JHU022 cells were treated with BME. RNA and protein expression were analysed for modulation of glycolytic and lipogenesis genes by quantitative real-time PCR, western blot analyses and immunofluorescence. Lactate and pyruvate level was determined by GC/MS. Extracellular acidification and glycolytic rate were measured using the Seahorse XF analyser. Shotgun lipidomics in Cal27 and JHU022 cell lines following BME treatment was performed by ESI/ MS. ROS was measured by FACS.

**Results:**

Treatment with BME on oral cancer cell lines significantly reduced mRNA and protein expression levels of key glycolytic genes SLC2A1 (GLUT-1), PFKP, LDHA, PKM and PDK3. Pyruvate and lactate levels and glycolysis rate were reduced in oral cancer cells following BME treatment. In lipogenesis pathway, we observed a significant reduction of genes involves in fatty acid biogenesis, ACLY, ACC1 and FASN, at the mRNA and protein levels following BME treatment. Further, BME treatment significantly reduced phosphatidylcholine, phosphatidylethanolamine, and plasmenylethanolamine, and reduced iPLA2 activity. Additionally, BME treatment inhibited lipid raft marker flotillin expression and altered its subcellular localization. ER-stress associated CHOP expression and generation of mitochondrial reactive oxygen species were induced by BME, which facilitated apoptosis.

**Conclusion:**

Our study revealed that bitter melon extract inhibits glycolysis and lipid metabolism and induces ER and oxidative stress-mediated cell death in oral cancer. Thus, BME-mediated metabolic reprogramming of oral cancer cells will have important preventive and therapeutic implications along with conventional therapies.

**Graphical abstract:**

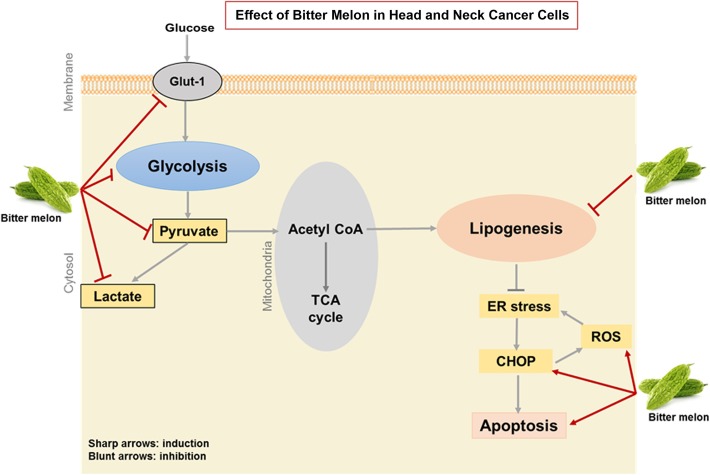

## Background

Oral squamous cell carcinoma represents one of the most common malignancy worldwide and estimated incidence rate of cancers in oral cavity and pharynxes is 53,000 and death rate is 10,860 in 2019 in the USA [1]. Oral cancers are highly heterogeneous, containing many genetic alterations rendering them refractory to specific targeted drugs. Traditional risk factors include tobacco smoking, alcohol consumption, betel nut chewing, human papillomavirus and genetic predisposition such as Fanconianemia [2]. Despite advancement of therapy and technology made over the past few decades, morbidity remains high due to lack of early detection markers, frequent association with metastasis, and lack of effective chemotherapeutic agents [3, 4]. With a five-year survival rate of approximately 50%, there is a critical clinical need to understand the disease process, and to identify better preventive and therapeutic strategies.

Many cancer cells display elevated glycolysis by converting glucose to lactate through pyruvate even in the presence of adequate oxygen, known as Warburg effect [5]. This effect minimizes oxidative stress, and promotes proliferation in cancer cells. On the other hand, normal cells efficiently produce energy through the conversion of glucose to pyruvate followed by TCA cycle and electron transport chain (ETC) in mitochondria. Avoiding ETC is advantageous for regulating reactive oxygen species (ROS) generation in cancer cells [6]. Excess pyruvates take part in de novo lipogenesis processes like producing fatty acids, phospholipids and cholesterol biosynthesis [7].

Like glycolysis, lipogenesis is enhanced in cancer cells compared to normal cells, and both pathways are linked. This adaptation favours cancer cells not only for energy production but also for formation of phospholipid bilayers, and protection against oxidative damage and stress induced cell death [8]. In oral cancer, increased *de-novo* fatty acid synthesis was noted and modulation in lipid metabolism has been implicated with increased invasiveness [9, 10]. Thus, from preventive and therapeutic perspective, it would be promising approach in research how best to target cancer metabolism.

We and others observed potential anticancer effects of bitter melon (*Momordica charantia*) extract (BME) against different cancer models including oral cancer by inhibiting cell proliferation, inducing apoptosis, modulating both cell signalling and immune systems [11–19]. Further, our transcriptome data and Gene Ontology analysis from BME mediated mouse tongue cancer prevention suggested a modulation in “metabolic process” [18]. This observation prompted us to investigate the role of BME at the molecular levels on glucose and lipid metabolism in oral cancer cells. In this study, we observed that BME treatment induces cell death by inhibiting glycolysis and lipogenesis pathways in oral cancer cells. To the best of our knowledge, this is the first study describing metabolic modification by BME in oral cancer.

## Methods

### Cell culture and preparation of bitter melon extract (BME)

HNSCC cell line Cal27 was purchased from the ATCC. JHU022 cell line was procured from the Johns Hopkins University. Cal27 and JHU022 cells were maintained in RPMI1640 media supplemented with 10% FBS and 1% penicillin/streptomycin in a humidified CO_2_ incubator. The cell lines are routinely tested in our laboratory to rule out mycoplasma contamination using commercial Lonza MycoAlert™ Mycoplasma Detection kit. Bitter melon extract (BME) was prepared from the Chinese variety of young bitter melons (raw and green) as discussed previously [13]. Briefly, BME was extracted from whole fruit without seeds using a household juicer at room temperature and centrifuged at 15000x g at 4 °C for 30 min. BME was stored at − 80 °C for further analysis. Cal27 cells were treated with 2% BME and JHU022 cells were treated with 3% BME and different analysis were performed. All the experiments were done at least in triplicate.

### RNA isolation and expression analysis

Cal27 and JHU022 cells were treated with/without BME for 30 h. Total RNA was extracted by TRIzol reagent followed by cDNA synthesis with SuperScript III Reverse Transcriptase (Life technology, USA). Real-time PCR was performed for quantitation of gene expression using specific primers (Table 1) by SYBR green based detection system. 18 s rRNA was used as an endogenous control. The relative gene expression was analysed by 2^-∆∆CT^ method. Each sample was loaded in triplicate.

**Table 1:** List of primes used in qRT-PCR

Human primers	Sequence
Hs GLUT1	Forward 5′- GGGGTCCTATAAACGCTACGG-3′
	Reverse 5′- GGGGGCATTGATGACTCCAG-3′
Hs HK1	Forward 5′-AGTTTGACAGGGAGATAGACC-3′
	Reverse 5′-CATCACTGGTGTTAAACTTCC-3′
Hs HK2	Forward 5′-AACAGCCTGGACGAGAGCAT-3′
	Reverse 5′-GCCAACAATGAGGCCAACTT-3′
Hs PFKP	Forward 5′- CAGAAGTACGCCTACCTCAAC-3′
	Reverse 5′- CTCCAGAACGAAGGTCCTCT-3′
Hs GPI	Forward 5′-GATGGTAGCTCTCTGCAGCC-3′
	Reverse 5′-GCCATGGCGGGACTCTTG-3′
Hs TPI	Forward 5′-AGGCATGTCTTTGGGGAGTC-3’
	Reverse 5′-AGTCCTTCACGTTATCTGCGA-3’
Hs ENO1	Forward 5′-CGCCTTAGCTAGGCAGGAAG-3’
	Reverse 5′-GGTGAACTTCTAGCCACTGGG-3’
Hs PKM	Forward 5′-CAGAGGCTGCCATCTACCAC-3’
	Reverse 5′-GGCCTTGCCAACATTCATGG-3’
Hs PDK3	Forward 5′- CCCCTTTGGCTGGATTTGGTTA-3’
	Reverse 5′- CACAGAGAGGACCACAGCATT-3’
Hs LDHA	Forward 5′-AGCTGTTCCACTTAAGGCCC-3’
	Reverse 5′-TGGAACCAAAAGGAATCGGGA-3’
Hs ACLY	Forward 5′-GACTTCGGCAGAGGTAGAGC-3’
	Reverse 5′-TCAGGAGTGACCCGAGCATA-3’
Hs ACC1	Forward 5′-TCACACCTGAAGACCTTAAAGCC-3’
	Reverse 5′- AGCCCACACTGCTTGTACTG-3’
Hs FASN	Forward 5′- GCAAGCTGAAGGACCTGTCT-3′
	Reverse 5′- TCCTCGGAGTGAATCTGGGT-3′
Hs 18S	Forward 5′-GTCATAAGCTTGCGTTGATT-3’
	Reverse 5′-TAGTCAAGTTCGACCGTCTT-3’

### Protein isolation and western blot analysis

Control or BME treated cell lysates were prepared, and western blot analysis was performed using specific antibodies to GLUT-1, PFKP, LDHA, PDK3, ACLY, FASN, and Flot-1 (Santa Cruz Biotechnology), ACC1 and CHOP (Cell Signaling Technology). Anti-mouse or anti-rabbit secondary antibodies were purchased from BIO-RAD. The blot was reprobed with actin-HRP antibody to compare protein load in each lane. Densitometry analysis was done using Image J software (NIH).

### Determination of lactate and pyruvate level by GC/MS

Cal27 and JHU022 cells were treated with or without BME for 30 h. Cells were washed in ice-cold PBS and rapidly quenched with 80% methanol. [^13^C_3_] lactate and [^13^C_3_] pyruvate (Cambridge Isotope Labs, Tewksbury, MA) were spiked into the samples as internal standards for quantitative analysis. Extracts were then sonicated and centrifuged at 14,000×g for 15 min at 4 °C. The clear supernatant was dried under a stream of nitrogen gas to complete dryness to clear precipitate and gas chromatography/mass spectrometry (GC/MS) experiment was performed, as described previously [20].

### Determination of extracellular acidification and glycolytic rate

The Cal27 and JHU022 cells (2 × 10^4^ cells/ well) were seeded into a 96 well-plate (Seahorse XF96 Cell Culture Microplates, Agilent) and treated with or without BME for 24–36 h. Cells were assessed for extracellular acidification rate (ECAR) and oxygen consumption rate (OCR) to understand glycolysis rate (ECAR/ OCR) using the Seahorse XF analyser (Agilent) as described previously [21].

### Lipidomics

Control or BME treated Cal27 and JHU022 cell suspensions were subsequently subjected to modified Bligh-Dyer extraction in the presence of lipid class internal standards, including heptadecanoic acid, 1,2-ditetradecanoyl-*sn*-glycero-3-phosphoethanolamine, heptadecanoyl cholesteryl ester, N-heptadecanoyl ceramide, and 1,2-dieicosanoyl-*sn*-glycero-3-phosphocholine [22]. For phospholipids, lipid extracts were diluted in methanol/chloroform (4/1, v/v), and molecular species were quantified by electrospray ionization-tandem mass spectrometry (ESI-MS/MS) on a triple quadrupole instrument (Thermo Fisher Quantum Ultra) using shotgun lipidomics. Phosphatidylcholine molecular species were quantified as sodiated adducts in the positive-ion mode using neutral loss scanning for 59.1 amu (collision energy = − 28 eV). Cholesteryl ester molecular species were quantified as sodiated adducts in the positive-ion mode using neutral loss scanning for 368.5 amu (collision energy = − 25 eV). Ceramide molecular species were quantified in the negative-ion mode using neutral loss scanning for 256.2 amu (collision energy = 32 eV). Phosphatidylethanolamine and plasmenylethanolamine molecular species were first derivatized to their fMOC species and then analysed by neutral loss scanning for 222.2 amu (collision energy = 30 eV) in negative ion mode (HAN). Individual molecular species were quantified by comparing the ion intensities of the individual molecular species to that of the lipid class internal standard, with additional corrections for type I and type II [13C] isotope effects.

### Calcium-independent phospholipase A2 (iPLA_2_) activity assay

Control or BME treated Cal27 and JHU022 cell was subjected to iPLA_2_ activity was measured as described previously [23]. Briefly, cells were washed with ice-cold PBS followed by PLA_2_ assay buffer, and PLA_2_ activity in the supernatant was measured at 37 °C for 5 mins, using 100 μM (16:0, [^3^H] 18:1) plasmenylcholine as substrate.

### Reactive oxygen species (ROS) analysis

Cal27 and JHU022 cells were treated with/without BME for 30 h. For mitochondrial ROS measurement cells were stained with MitoSOX (Molecular Probes, Invitrogen) at 5 μM for 40 min at 37 °C and flow cytometry analysis was performed as described previously [24].

### Immunofluorescence analysis

Control and BME treated cells were fixed with chilled methanol for 5 min at -20 °C. After blocking with 5% BSA, primary antibody to Flotillin was added for overnight at 4 °C, and anti-mouse immunoglobulin conjugated to Alexa Fluor 647 (Molecular Probes) for 1 h at room temperature. Cells were counter stained with DAPI (4′,6′-diamidino-2-phenylindole) for nuclear staining. Two-channel optical images (red and blue) were collected using the sequential scanning mode of the Olympus FV1000 confocal system. The images were merged digitally to monitor co-localization in which two different colors produce a distinct color, whereas physically separate signals retain their individual colors.

### Statistical analysis

The results are expressed as means ± standard errors of the means. Student’s t test was used for comparisons of two groups (control and BME-treated). *P*-values less than 0.05 was considered statistically significant. All experiments were repeated at least three times, and representative data are shown.

## Results

### BME treatment modulates expression of glycolytic genes

Metabolic reprogramming is one of the hallmarks of cancers including oral cancer. We recently observed that BME treatment prevented tobacco associated carcinogen 4-Nitroquinoline 1-oxide (4NQO) induced mouse tongue cancer [18]. Our transcriptome analysis using next-generation RNA-seq from the tongue lesions with or without BME treatment and subsequent Gene Ontology analysis revealed several biological processes modulated significantly in BME fed group compared to the cancer. Among them, we identified “Metabolic process (GO: 0008152)” where several key regulatory genes of glycolysis pathway were modulated between cancer group and BME treated group (Table 2).

**Table 2:** Transcriptomic data of glycolysis and lipogenesis pathways in 4NQO induced mouse tongue cancer group and in BME fed group

Genes	Cancer compared to normal (Fold)	BME compared to cancer (Fold)
Glucose transporter-1 (Glut1/SLC2A1)	8.13	−2.9***
Hexokinase-1 (HK1)	1.3	−1.35*
Hexokinase-2 (HK2)	1.4	1.07
Phosphofructo kinase (platelet) (PFKP)	1.8***	−1.4
Phosphogluco isomerase (GPI)	−1.04	1.06
Triosphosphate isomerase (TPI)	−1.1	−1.1
Enolase- 1 (ENO-1)	3.08***	−1.8*
Pyruvate kinase muscle (PKM)	1.2	−1.3
Pyruvate dehydrogenase kinase 3 (PDK3)	1.7**	−1.1
Lactate dehydrogenase alpha (LDHA)	1.9*	−1.5
ATP Citrate Lyase (ACLY)	1.2	−1.3*
Acetyl-CoA Carboxylase 1 (ACC1)	1.02	−1.33
Fatty Acid Synthase (FASN)	1.11	−1.5*

* *P* < 0.05; ** *P* < 0.01; ***P < 0.001

**Fig. 1:**
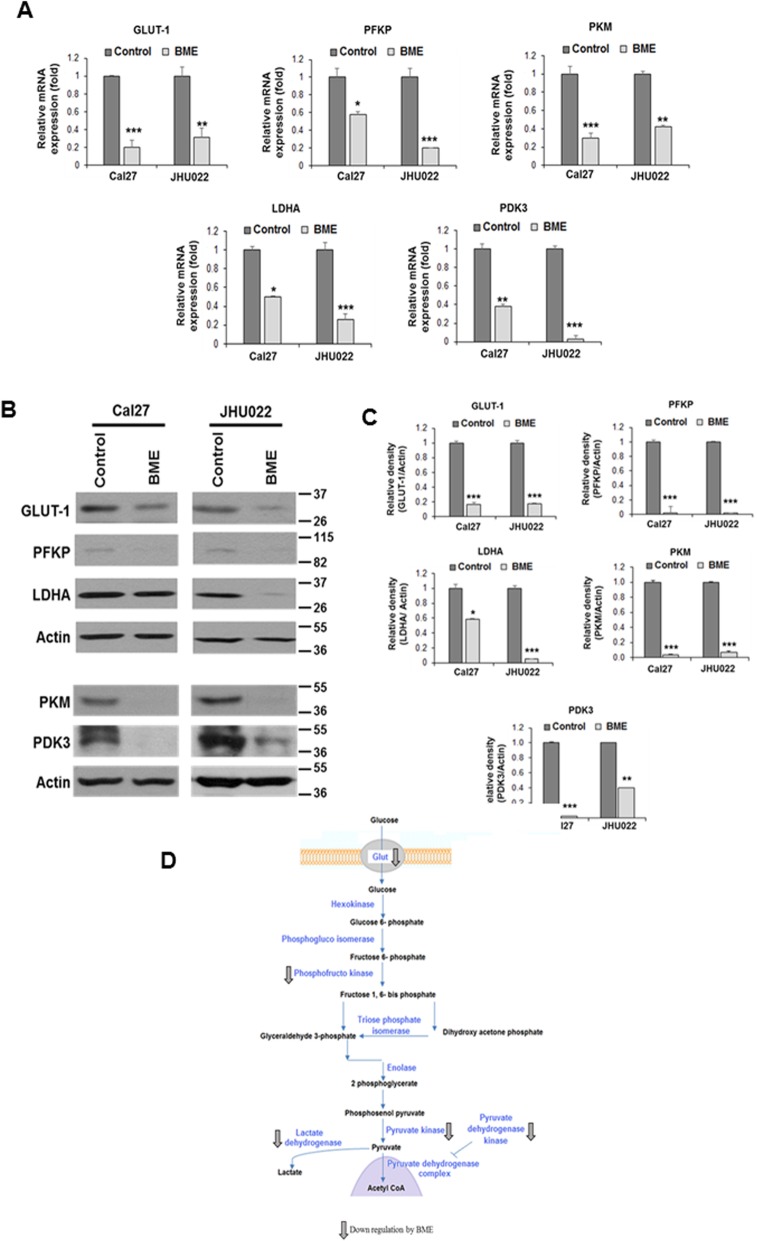
BME treatment reduces expression of glycolytic genes. **a:** Relative mRNA expression of GLUT-1, PFKP, PKM, LDHA, and PDK3 was analysed by q-RT-PCR in Cal27 and JHU022 cells with/without BME. 18 s gene was used as internal control. **b:** Cell lysates from Cal27 and JHU022 with or without BME treatment for 30 h were subjected to Western blot analysis for GLUT-1, PFKP, LDHA, PKM and PDK3 using specific antibodies. The membrane was reprobed with antibody to actin as an internal control. **c:** Quantitative of Western blot band intensities using Image-J software. Small bar indicates standard error (*, *p* < 0.05; **, *p* < 0.01; *** *p* < 0.001). **d:** Schematic diagram showing different genes regulate glycolysis and effect of BME on the genes

We next examined changes in glycolysis genes following BME treatment in human oral cancer cell lines, Cal27 and JHU022. We observed a significant down-regulation of glucose transporter SLC2A1 (GLUT-1) mRNA expression in both the cell lines following BME treatment (Fig. 1a). In the glycolytic pathway, phosphofructokinase 1 (PFK1) catalyses one of the key regulatory steps that converts fructose 6-phosphate to fructose 1, 6-bisphosphate. PFK1 has 3 isoforms: platelet (PFKP), muscle (PFKM), and liver (PFKL). PFKP is a major isoform of cancer specific PFK1 [25]. A significant down-regulation of PFKP was observed in Cal27 and JHU022 cells following BME treatment (Fig. 1a). Pyruvate kinase (PK) catalyzes the last step in glycolysis, the conversion of pyruvate from phosphoenolpyruvate. Mammals have four PK isoforms (L, R, M1 and M2) among these pyruvate kinases (muscle), PKM1 and PKM2, are alternative spliced products of PKM and overexpressed in cancers [26]. The PKM2 is involved in head and neck cancer initiation and progression by promoting cell proliferation and migration, and inhibiting apoptosis [27]. Significant down-regulation of PKM was seen following BME treatment (Fig. 1a). Lactate dehydrogenase-A (LDHA) facilitates glycolysis by converting pyruvate to lactate. In the oral cancer cell lines, we observed a significant reduction of these gene following BME treatment (Fig. 1a). Pyruvate dehydrogenase kinase (PDK) is a mitochondrial enzyme that is activated in a variety of cancers, resulting in the selective inhibition of pyruvate dehydrogenase, a complex of enzymes that converts cytosolic pyruvate to mitochondrial acetyl-CoA, the substrate for the TCA cycle [28]. PDKs have four isoforms and a significant up-regulation of PDK3 was seen in cancer in our RNA-Seq data. We observed significant down-regulation of PDK3 following BME treatment (Fig. 1a). We did not observe a significant change in other glycolysis genes such as hexokinase (HK-1 and 2), phosphoglucoisomerase (GPI), triosephosphateisomerase (TPI) and enolase-1 (ENO-1) in our experimental system (data not shown). Further, we observed a significant reduction of GLUT-1, PFKP, PKM, LDHA and PDK3 protein expression in Cal27 and JHU022 cells following BME treatment (Fig. 1b and c). Thus, our result demonstrated that BME treatment in oral cancer cell lines impaired glycolysis by inhibiting expression of key glycolytic genes (Fig. 1d). Similar effects were observed in two other oral cancer cell lines, JHU029 and MDA1386 cells (data not shown). Subsequent analysis was performed using Cal27 and JHU022 cells.

### BME inhibits pyruvate/lactate production and glycolysis rate

**Fig. 2:**
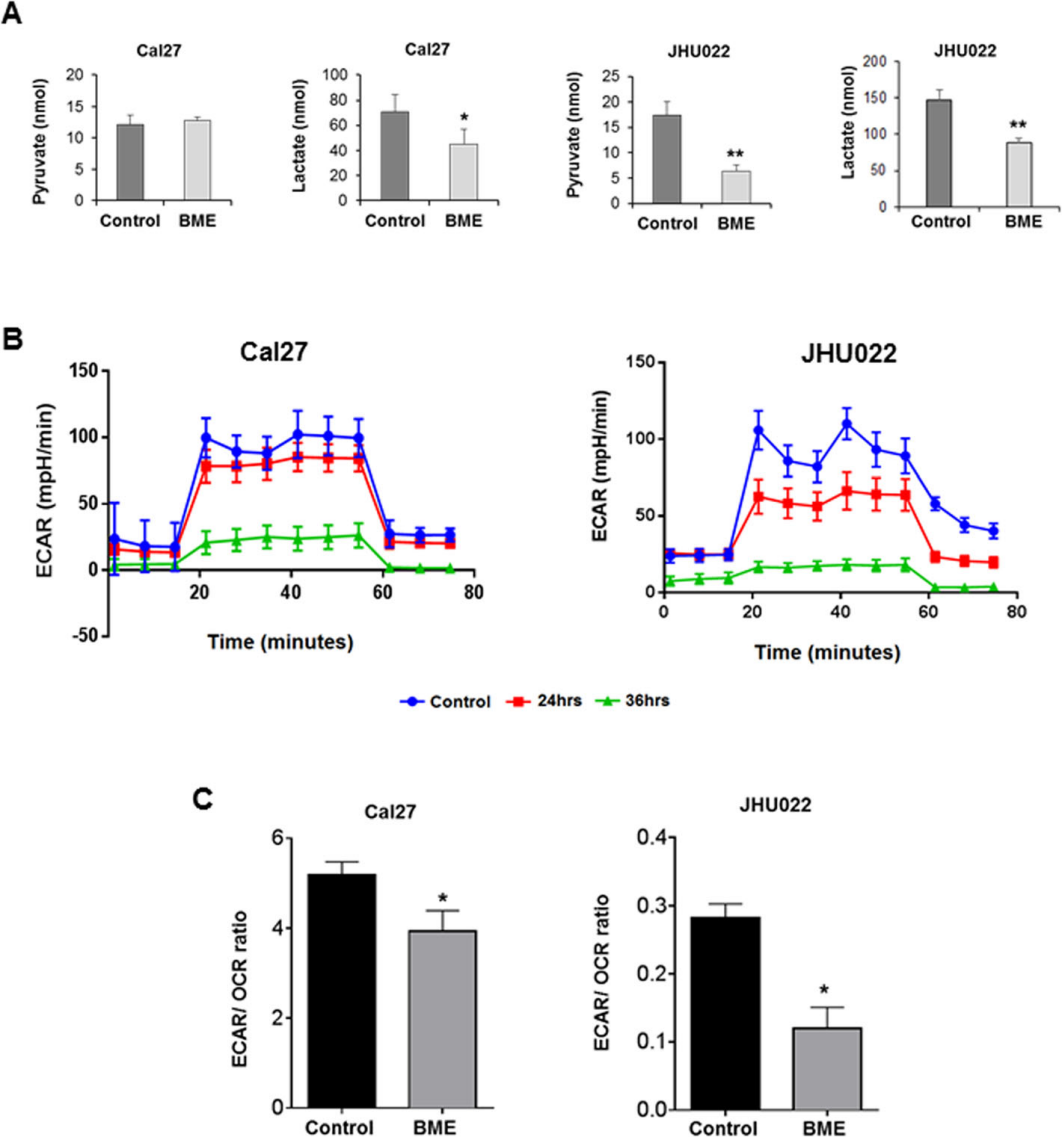
BME treatment reduces pyruvate and lactate and glycolysis rate. **a:** Cal27 and JHU022 cells were treated with BME for 30 h and subjected to GC/MS analysis to determine levels of pyruvate and lactate. **b:** Cal27 and JHU022 cells were treated with BME for 24 h and 36 h. Extracellular acidification rate (ECAR) (glycolysis) was assessed using the Seahorse XF extracellular flux analyser. **c:** Relative glycolysis rate ECAR/OCR (oxygen consumption rate) was calculated at 36 h. Small bar indicates standard error (*, *p* < 0.05; **, *p* < 0.01; *** *p* < 0.001)

Since we observed reduced expression of glycolytic genes by BME treatment, we analysed pyruvate and lactate production, two key metabolites of glycolysis pathway. Gas chromatography/mass spectrometry (GC/MS) analysis revealed significant reduction of pyruvate in JHU022 cells (Fig. 2a). On the other hand, lactate level was reduced significantly in both the cell lines following BME treatment. To further investigate the glycolytic rate and its outcome, extracellular acidification rate (ECAR) and mitochondrial oxygen consumption rate (OCR) were measured. A significant reduction of glycolysis (ECAR) and relative glycolysis rate (ECAR/ OCR ratio) in both Cal27 and JHU022 cells following BME treatment was observed (Fig. 2b and c). Together, our results suggested that BME treatment preferentially reduced glycolytic metabolism.

### BME treatment modulates lipid metabolism

**Fig. 3:**
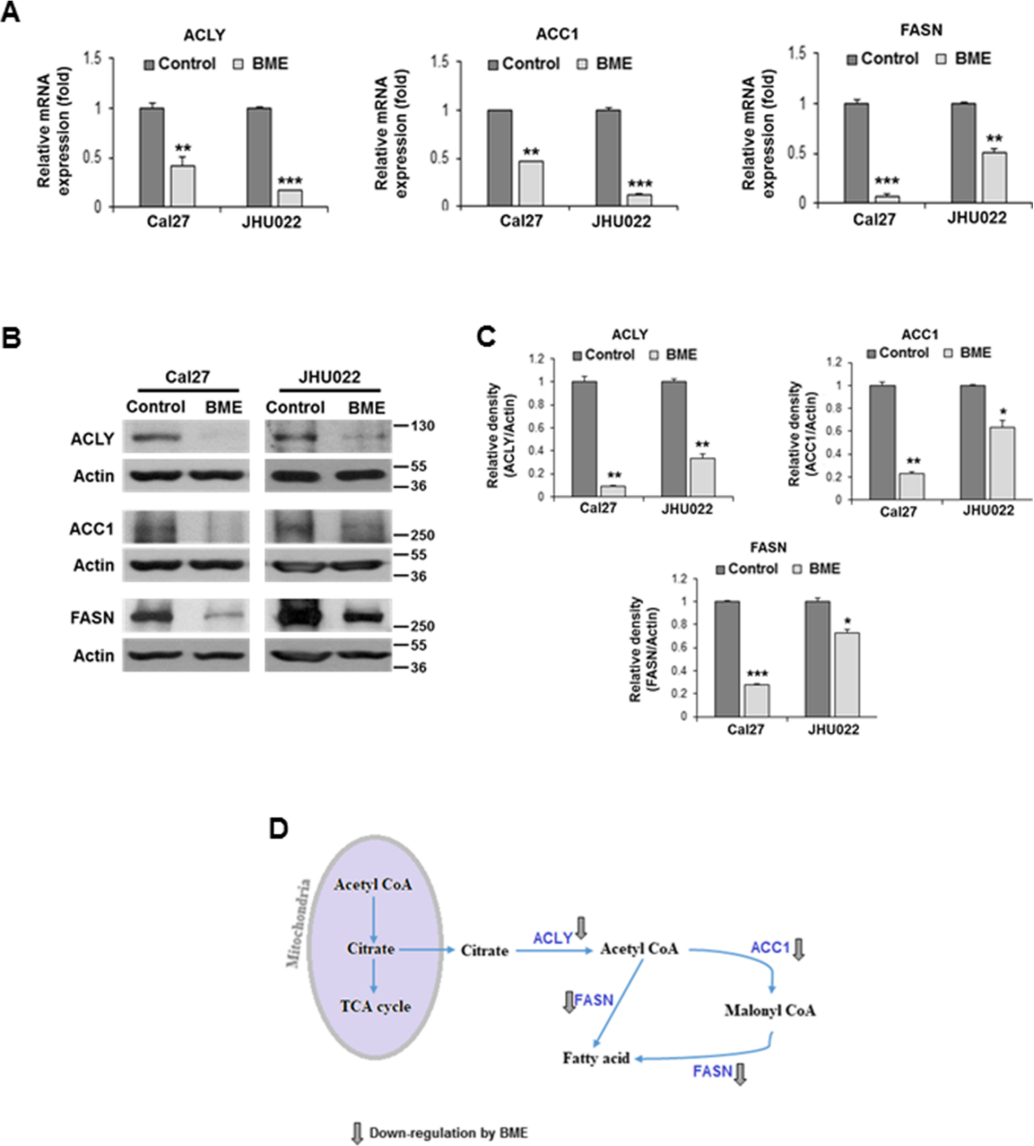
BME treatment inhibits expression of different enzymes of de novo lipogenesis. **a:** Relative mRNA expression of ACLY, ACC1 and FASN analysed by qRT-PCR in Cal27, and JHU022 cells with or without BME for 30 h. 18 s gene was used as internal control. **b:** Cell lysates from Cal27 and JHU022 with or without BME treatment for 30 h were subjected to Western blot analysis for ACLY, ACC1 and FASN using specific antibodies. The membrane was reprobed with antibody to actin as an internal control. **c:** Quantitative of Western blot band intensities using Image-J software. Small bar indicates standard error (*, *p* < 0.05; **, *p* < 0.01; *** *p* < 0.001). **d:** Schematic diagram showing different genes regulate lipogenesis and effect of BME on the genes

We also observed down-regulation of several lipogenesis genes in RNA-seq data (Table 2). We therefore, analysed these gene expression in Cal27 and JHU022 cells following BME treatment. We observed a significant down-regulation of mRNA expression of ATP citrate lyase (ACLY), acetyl CoA carboxylase 1 (ACC1) and fatty acid synthase (FASN) genes following BME treatment (Fig. 3a). Western blot analysis also showed a significant reduction in protein expression level (Fig. 3b and c). The ACLY catalyses the conversion of citrate to cytosolic acetyl-CoA linking increased glycolysis to enhanced lipogenesis [29]. The ACC1 and FASN catalyse fatty acid synthesis from the acetyl CoA. Our results suggested that BME treatment reduces ACLY, ACC1 and FASN in modulation of de novo lipogenesis (Fig. 3d).

**Fig. 4:**
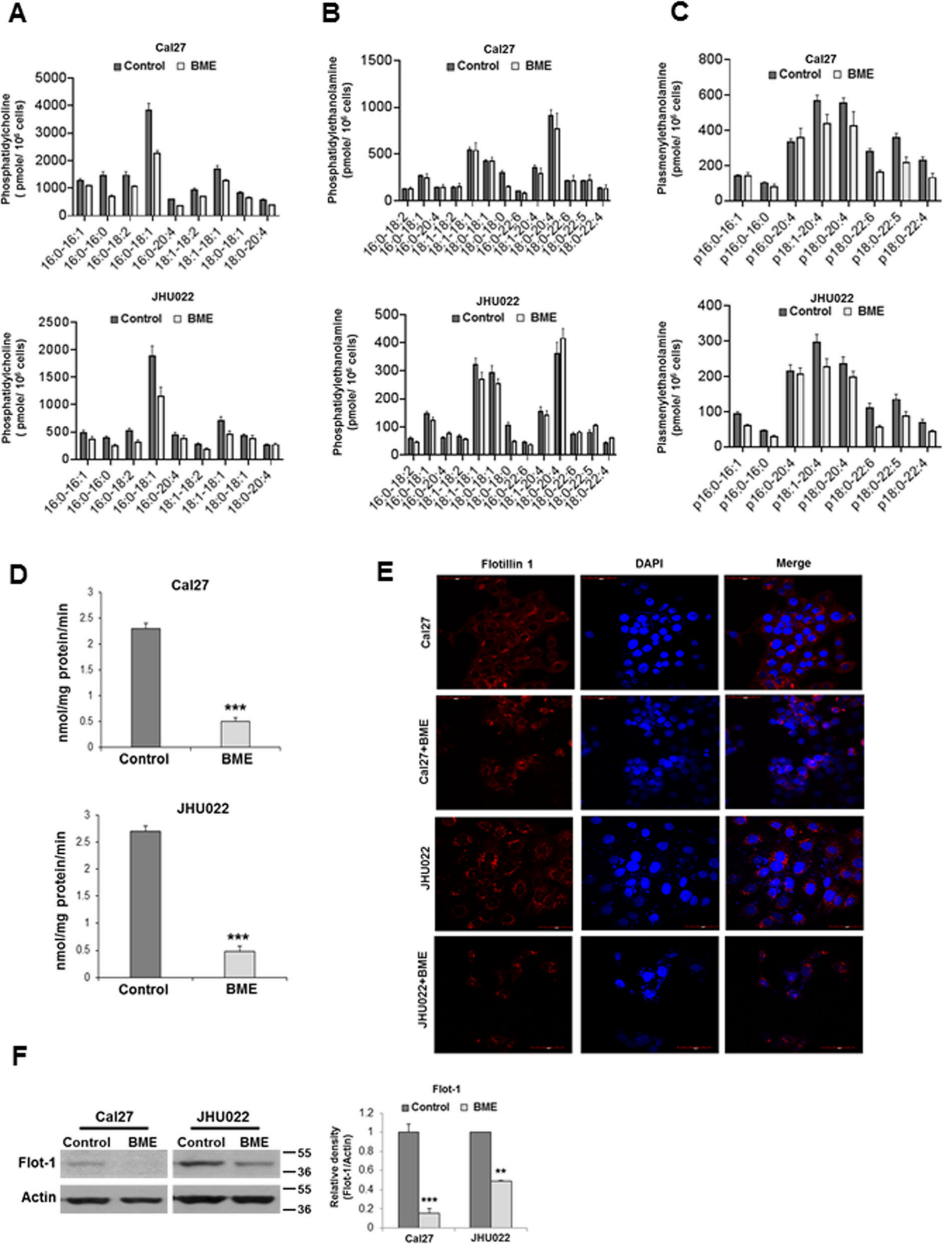
BME treatment inhibits phospholipids, iPLA2 activity and lipid raft. Cal27 and JHU022 cells were treated with BME for 30 h and lipid profile was analysed by electrospray ionization-tandem mass spectrometry (ESI-MS/MS). Representative mass- spectra showing **a:** phosphatidylcholine (PC), **b:** phosphatidylethanolamine (PE), and **c**: plasmenylethanolamine. **d:** Cal27 and JHU022 cells were treated with BME for 30 h and intracellular iPLA2 activity was assayed. Small bar indicates standard error (*, p < 0.05; **, p < 0.01; *** p < 0.001). **e:** Cal27 and JHU022 cells were treated with BME for 30 h and stained with antibody to Flotillin (red) and DAPI (blue). Representative confocal microscopic images showing reduced expression of Flotillin in BME treated cells compared to control cells. Magnifications 60X and scale bar 50 μm. **f:** Cell lysates from Cal27 and JHU022 with or without BME treatment for 30 h were subjected to Western blot analysis for Flotillin-1 using specific antibody. The membrane was reprobed with antibody to actin as an internal control. Quantitative of Western blot band intensities using Image-J software (shown in right). Small bar indicates standard error (**, *p* < 0.01; *** *p* < 0.001)

Next, we performed shotgun lipidomics in Cal27 and JHU022 cell lines following BME treatment by ESI/ MS. We observed a reduction of several molecular species of phosphatidylcholine (PC) and phosphatidylethanolamine (PE) specifically plasmelylethanolamine (pPE) following BME treatment (Fig. 4a, b and c). PC and PE are most abundant phospholipids in membrane of mammalian cells and subcellular organelles, and cancer cells generally convert free fatty acids for phospholipid synthesis [30]. However, we did not see a change in ceramides and cholesteryl esters level following BME treatment (data not shown). We observed inhibition in fatty acid synthesis genes and reduction in PC, PE and pPE by BME, we therefore examined consequences of this event. Calcium-independent phospholipase A_2_ (iPLA_2_) is ubiquitously expressed in mammalian cells and participates in several biological processes including lipid metabolism, phospholipid remodelling, cell differentiation, maintenance of mitochondrial integrity, cell proliferation, signal transduction, and cell death [31]. iPLA_2_ hydrolyses phospholipids to release free fatty acid, arachidonic acid, and lysophospholipid [23]. We observed significant reduction of iPLA_2_ activity following BME treatment (Fig. 4d).

Since we observed modulation in phospholipids and fatty acids synthesis, we hypothesised that BME treatment might modulate lipid raft. Lipid raft is cell signalling hub and containing different proteins [32]. Flotillins are one of the key components of lipid raft [32, 33]. There are two members, flotillin-1 (Flot-1) and flotillin-2 (Flot-2), that stabilize each other by forming a hetero-oligomer. High expression of flotillins was evident in cancers and associated with tumor growth, metastasis and poor prognosis [33]. We examined the flotillin expression and localization by immunofluorescence in Cal27 and JHU022 cells treated with BME. We observed increased flotillin expression in control Cal27 and JHU022 cells and located all over the cells (Fig. 4e). On the other hand, BME treated cells display reduced flotillin expression and a discrete localization in the perinuclear region (Fig. 4e). We also observed lower flotillin-1 expression in BME treated cells (Fig. 4f), suggesting BME treatment inhibits lipid rafts in oral cancer cells. Together, our results suggest an impairment of lipogenesis following BME treatment in oral cancer cell lines.

### BME treatment enhances CCAAT/enhancer-binding protein-homologous protein (CHOP) expression and reactive oxygen species (ROS) generation

**Fig. 5:**
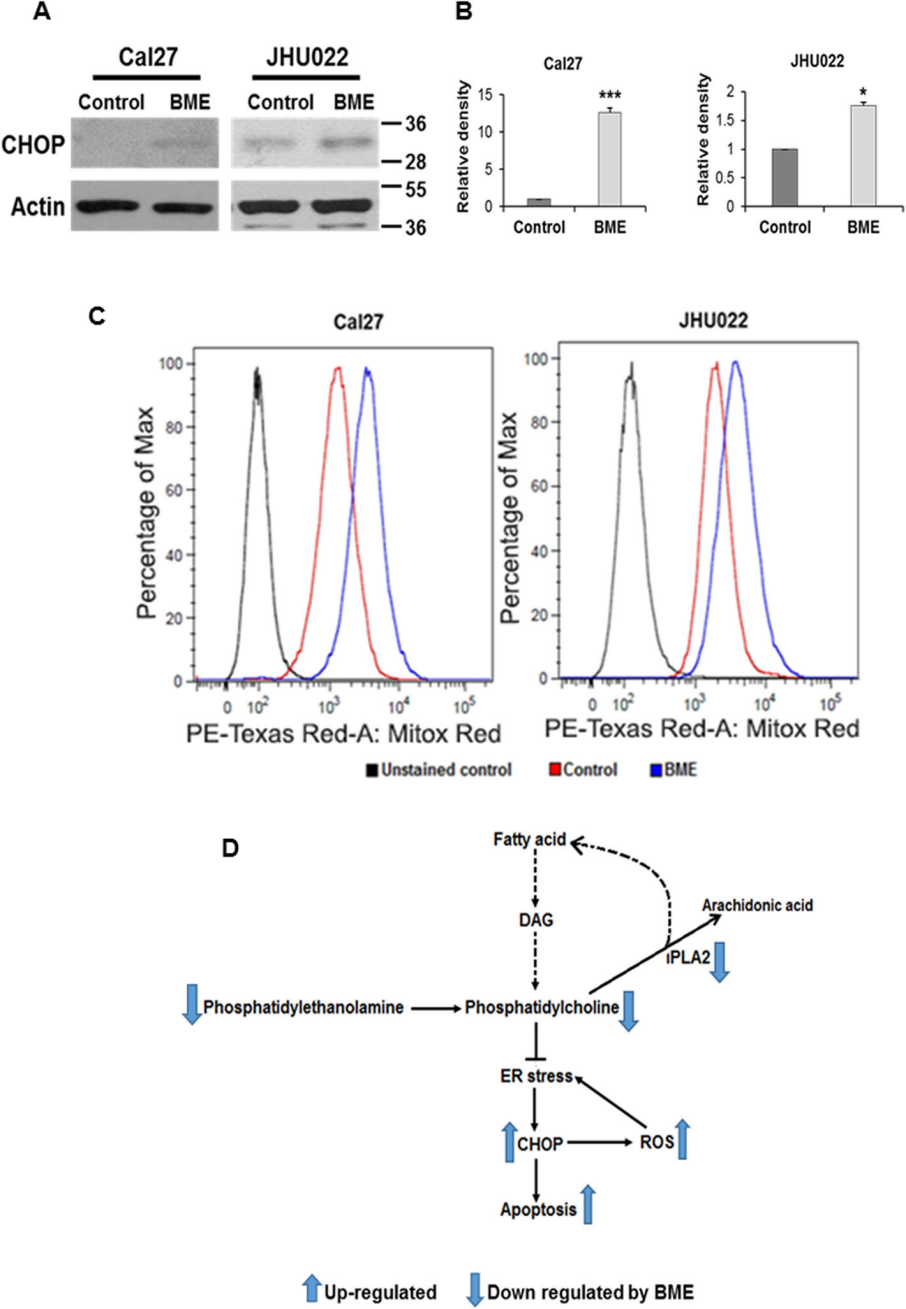
BME treatment induces CHOP and ROS generation. **a:** Cell lysates from Cal27 and JHU022 with/without BME treatment were subjected to Western blot analysis for the CHOP using specific antibodies. The membrane was reprobed with antibody to actin as an internal control. **b:** Quantitative of Western blot band intensities using Image-J software. Small bar indicates standard error (*, *p* < 0.05; **, *p* < 0.01; *** *p* < 0.001). **c:** Cal27 and JHU022 with or without BME treatment were stained with mitoSox and flow cytometric analysis was performed to analyse mitochondrial ROS level at 510 nm. **d:** Schematic representation of lipogenesis pathway and probable mode of action of BME on regulation of phospholipids and thereby modulation ER stress and ROS associated cell death

We reported previously that BME treatment induces apoptosis in oral cancer cell lines [13], although mechanism is poorly understood. BME mediated modulation in lipid metabolism via inhibition of phospholipid biogenesis can induce endoplasmic reticulum (ER) stress and ROS generation. Following ER stress, CHOP is activated and induces caspase mediated cellular apoptosis [34]. To investigate this possibility, we examined expression of pro-apoptotic CHOP in Cal27 and JHU022 cells with or without BME treatment. We observed a significant induction of CHOP protein following BME treatment in these cell lines (Fig. 5a and b). ER stress can also induce mitochondrial ROS level and increased mitochondrial ROS can enhance ER stress by feedback mechanism [35]. We also observed increased mitochondrial ROS level in BME treated cells (Fig. 5c). Our results suggest that modulation of de novo lipogenesis, induction of ER stress and ROS leads to apoptotic cell death by BME (Fig. 5d).

## Discussion

The metabolic profiles of cancer cells are different from normal cells due to the aerobic glycolysis (Warburg effect) and lipogenesis, key metabolic pathways which drive cancer progression [21]. Thus, targeting these glycolysis and lipogenesis pathways may have broad range of implication in treatment of cancers. In the present study, we demonstrated that BME modulates glucose and lipid metabolism in oral cancer cells. The metabolic reprogramming is facilitated by BME through inhibition of (i) key regulatory genes of glycolysis pathway resulting reduction in pyruvate and lactate production as well as glycolysis rate, (ii) key fatty acid synthesis genes, reduction in phospholipids, inactivation of iPLA2, inhibition of lipid raft, and (iii) induction of ER stress and ROS mediated cancer cell death.

In both normal and cancer cells, glucose is an important source of energy and carbon. Glucose transporter GLUT-1 is first characterized transporter and over expression of GLUT-1 was seen in oral cancer [36]. Increased expression of GLUT-1 is associated with tumor stage, recurrence, poor patient survival and drug resistance [36, 37]. Different pre-clinical and clinical studies showed that natural products genistein and silybin targets GLUT-1, resulting in inhibition of glucose uptake, alteration in metabolism and induction of apoptosis [38, 39]. Activation of PFKP was reported in different cancers and targeted inhibition of PFK by azole derivative Clotrimazole showed inhibition in cancer cell proliferation, migration and glucose metabolism [40, 41]. PKM2 overexpression is implicated as prognostic markers and PMK2 inhibitors suppress cell growth [42]. LDHA, another important glycolytic gene, is up-regulated in many cancers including oral cancer, and regulates cancer cell proliferation, metastasis, angiogenesis and immune escape [43]. Inhibitors of LDHA showed anti-cancer effect in different pre-clinical models, although clinical trials are halted due to low permeability or non-specific toxicity. Upregulation of pyruvate dehydrogenase kinase (PDK) isoforms including PDK3 was reported in different cancers and associated with chemoresistance. PDK inhibitor dichloroacetate showed potential anticancer effect in different cancers including head and neck [39, 44] and in the clinical trials. In fact, the past few decades, the Warburg effect has been extensively investigated for targeted therapies to cancer cells, however success is limited due to toxicity or non-specific affect [43]. However, we did not observe any systemic toxicity using BME in our preclinical studies [16, 18]. Thus, inhibiting the expression of glycolytic genes by BME in oral cancer cells suggests its potential therapeutic efficacy. While we are ready to submit our manuscript, Dhar et al. [45] reported that bitter melon treatment in pancreatic cancer modulates lactate efflux and glucose metabolism, in agreement in our observation.

Increased glycolysis results in accumulation of lactic acid in tumor microenvironment. Accumulation of lactic acid maintains relatively low pH in microenvironment and helps cancer cells to escape immune destruction [46]. High lactic acid production is associated with oral cancer metastasis and radiation resistance [47, 48]. On the other hand, excess pyruvate facilitates either mitochondrial TCA cycle-electron transport chain or lipogenesis. Cancer cells generally prefer lipogenesis to avoid ETC and for producing energy, maintaining membrane integrity, preventing ROS and stress induced damage [6, 7]. Enhanced expression of lipogenic enzymes, such as ACLY, ACC1 and FASN, represent a nearly-universal phenotypic alteration in most tumors [49]. Therapeutic targeting of these enzymes showed potential anti-cancer effect in various pre-clinical models [49]. However, inhibitors of these targets displayed adverse side effects. We observed down-regulation of lipogenesis pathway by BME treatment in oral cancer cells. Our lipidomics data suggested a significant inhibition of several molecular species of PC, PE and pPE following BME treatment on oral cancer cells. Ethanolamine glycerophospholipids (pPE and PE) are the second most abundant phospholipid in mammalian cellular membranes, accounting for approximately 20% of the total phospholipids [50]. Translocation and redistribution of PE occurs during several distinct biological processes including cell death [50]. Several membrane-active peptides and small molecules, which bind to PE, exert cytotoxic activity by inducing cell death, and have the potential for development of a novel class of drugs based on their molecular scaffold.

Calcium-independent PLA_2_ (iPLA_2_) enzymes hydrolyse membrane phospholipids to produce free fatty acid and lysophospholipid [23]. Further, iPLA_2_ inhibition showed potential anti-inflammatory and anti-cancer property by inducing cell cycle arrest and apoptosis in different cancers [51, 52]. Inhibition of phospholipids and fatty acids synthesis modulate membrane lipid raft which is major receptor mediated signalling hub [32]. BME mediated inhibition of lipid raft (delocalization of flotillin) indicated its molecular mechanism of inhibition cell signalling. Lipid raft marker flotillin is associated with tumor growth, metastasis and poor prognosis [33]. Inhibition of phospholipid biogenesis could impair with membrane trafficking, lipid raft composition, ER stress and ROS generation [34, 49]. In our study, we observed induction of CHOP following BME treatment. CHOP is activated by ER stress and facilitates caspase mediated apoptosis [34].

Cancer metabolism is an emerging field and refers to a common set of metabolic changes that accompany neoplasm. The excessive aerobic glycolysis, known as Warburg effect, is a hallmark of cancer and is supported by several observations [53, 54]. BME does not exert anti-proliferative affect in normal cells [11]. We observed higher expression of glycolysis and lipid pathway genes in head and neck cancer cells compared to normal human oral keratinocytes cells (data not shown). We recently reported a higher expression of some key glycolysis and lipid metabolism genes in 4NQO induce mouse tongue tumor compared to normal tongue [18]. BME treatment reduced their expression to normal levels during prevention of carcinogenesis (Table 2). Further, inhibition of glucose and lipid metabolisms induce stress and apoptotic cell death [7, 49, 50]. Additionally, mitochondria play a dynamic role in cancer cells and targeting mitochondrial biogenesis affects cancer cell proliferation [54]. BME treatment in head and neck cancer cells enhances mitochondrial ROS production. Therefore, we have demonstrated BME mediated cell death is occurring by targeting multiple genes within these pathways. In-depth mechanism of the BME mediated modulation of these molecules remains to be elucidated.

## Conclusions

In summary, we demonstrated that BME treatment modulates glucose and lipid metabolism on oral cancer by modulating several key molecules. Aiming the core metabolic pathways, Warburg effect and lipogenesis, will have beneficial effect, and become promising preventive and therapeutic target for oral cancer patients in future therapies. Therefore, BME has high potential as a therapeutic agent against oral cancer.

## Data Availability

Data generated in this study are included in the article and raw data will be available upon request.

## Abbreviations

ACC1: Acetyl-CoA Carboxylase 1
ACLY: ATP Citrate Lyase
BME: bitter melon extract
CHOP: CCAAT/enhancer-binding protein-homologous protein
FASN: Fatty Acid Synthase
GLUT-1: glucose transporter protein type 1
iPLA_2_: calcium-independent phospholipase A2
LDHA: Lactate Dehydrogenase A
PC: phosphatidylcholine
PDK3: Pyruvate Dehydrogenase Kinase 3
PE: phosphatidylethanolamine
PFKP: Phosphofructokinase, Platelet
PKM: Pyruvate Kinase, Muscle
pPE: plasmenylethanolamine
ROS: reactive oxygen species
SLC2A1: Solute Carrier Family 2 Member 1
