# Correction to: Inhibition of the key metabolic pathways, glycolysis and lipogenesis, of oral cancer by bitter melon extract

**DOI:** 10.1186/s12964-019-0475-7

**Published:** 2019-11-19

**Authors:** Subhayan Sur, Hiroshi Nakanishi, Colin Flaveny, Joseph E. Ippolito, Jane McHowat, David A. Ford, Ratna B. Ray

**Affiliations:** 1 Department of Pathology, Saint Louis University, 1100 South Grand Boulevard, St. Louis, MO 63104 USA; 2 Department of Pharmacology and Physiology, Saint Louis University School of Medicine, St. Louis, MO USA; 3 Mallinckrodt Institute of Radiology, Washington University in Saint Louis, School of Medicine, Saint Louis, MO USA; 4 Biochemistry and Molecular, Biology, Saint Louis University, Saint Louis, MO USA

**Correction to: Cell Commun Signal**

**https://doi.org/10.1186/s12964-019-0447-y**

Following publication of the original article [1], it was reported that Fig. 1c was not entirely readable due to overlapping Fig. 1d. The publishers apologise for this error.

The updated Fig. 1 is supplied below. The original article [1] has been corrected.

**Fig. 1:**
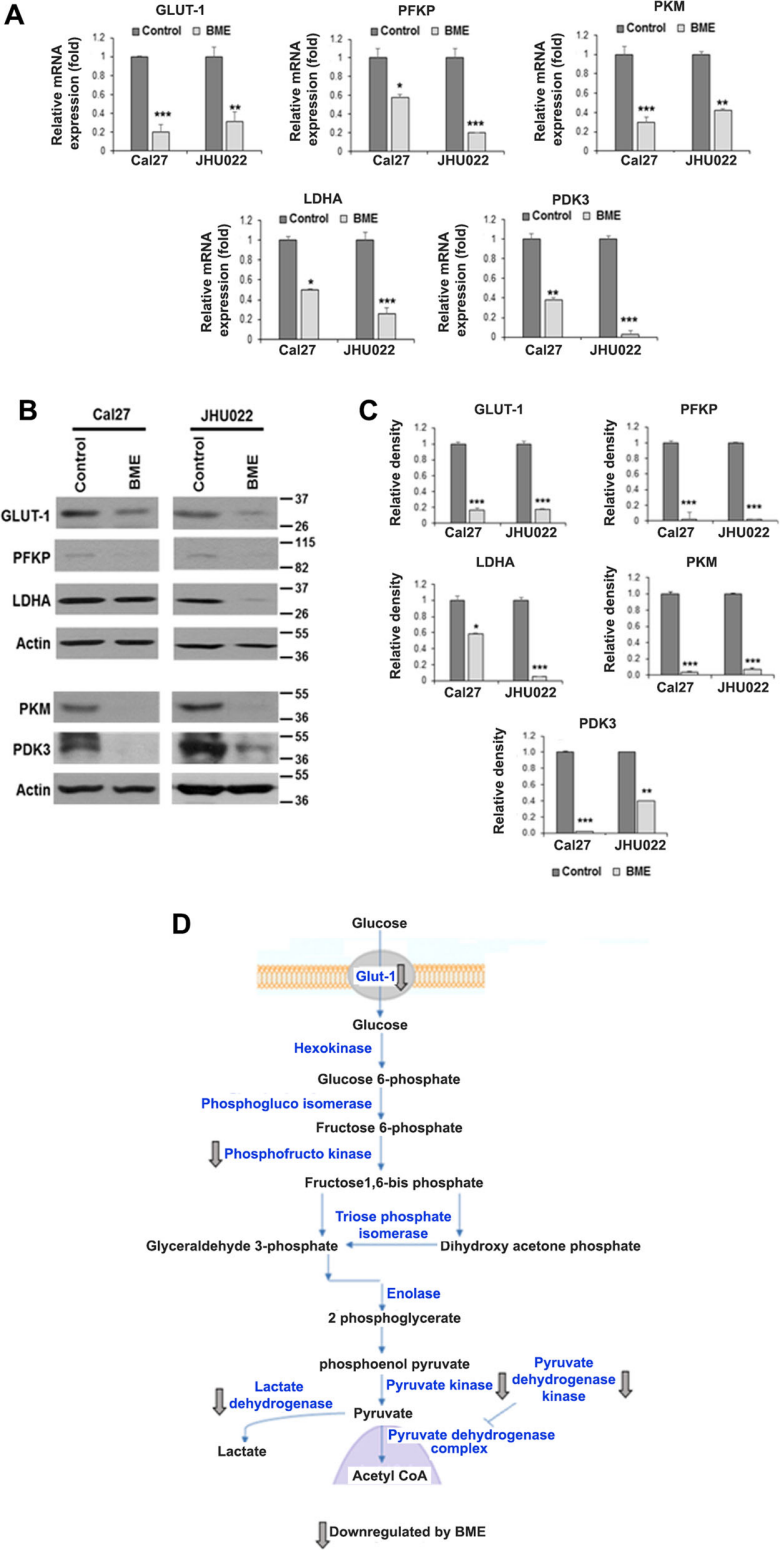
BME treatment reduces expression of glycolytic genes. **a:** Relative mRNA expression of GLUT-1, PFKP, PKM, LDHA, and PDK3 was analysed by q-RT-PCR in Cal27 and JHU022 cells with/without BME. 18 s gene was used as internal control. **b:** Cell lysates from Cal27 and JHU022 with or without BME treatment for 30 h were subjected to Western blot analysis for GLUT-1, PFKP, LDHA, PKM and PDK3 using specific antibodies. The membrane was reprobed with antibody to actin as an internal control. **c:** Quantitative of Western blot band intensities using Image-J software. Small bar indicates standard error (*, *p* < 0.05; **, *p* < 0.01; *** *p* < 0.001). **d:** Schematic diagram showing different genes regulate glycolysis and effect of BME on the genes
